# Computing optimal factories in metabolic networks with negative regulation

**DOI:** 10.1093/bioinformatics/btac231

**Published:** 2022-06-27

**Authors:** Spencer Krieger, John Kececioglu

**Affiliations:** Department of Computer Science, The University of Arizona, Tucson, AZ 85721, USA; Department of Computer Science, The University of Arizona, Tucson, AZ 85721, USA

## Abstract

**Motivation:**

A *factory* in a metabolic network specifies how to produce target molecules from source compounds through biochemical reactions, properly accounting for reaction stoichiometry to conserve or not deplete intermediate metabolites. While finding factories is a fundamental problem in systems biology, available methods do not consider the number of reactions used, nor address negative regulation.

**Methods:**

We introduce the new problem of finding optimal factories that use the *fewest reactions*, for the first time incorporating both first- and second-order *negative regulation*. We model this problem with directed hypergraphs, prove it is NP-complete, solve it via mixed-integer linear programming, and accommodate second-order negative regulation by an iterative approach that generates next-best factories.

**Results:**

This optimization-based approach is remarkably *fast in practice*, typically finding optimal factories in a few seconds, even for metabolic networks involving tens of thousands of reactions and metabolites, as demonstrated through comprehensive experiments across all instances from standard reaction databases.

**Availability and implementation:**

Source code for an implementation of our new method for *optimal factories* with negative regulation in a new tool called Odinn, together with all datasets, is available free for non-commercial use at http://odinn.cs.arizona.edu.

## 1 Introduction

Metabolic networks consist of all physical and biochemical reactions that occur within an organism or cell. They critically guide research in many areas, including metabolic engineering, where cell metabolism is altered to improve a targeted cell function, and interrogation of multi-species relationships, where the interaction between different organisms is determined by their metabolic output (Oberhardt *et al.*, 2009). Participants in these reactions have associated stoichiometric ratios, and the reactions are often catalyzed by an enzyme (positive regulation), or inhibited by a substrate (negative regulation). Metabolic networks, and closely-related cell-signaling networks, are traditionally represented by ordinary graphs (Sharan and Ideker, 2006; Vidal *et al.*, 2011); however, ordinary graphs cannot adequately model multiway reactions among compounds (Klamt *et al.*, 2009; Ritz *et al.*, 2014), and are unable to represent positive and negative regulation.

Recently, directed *hypergraphs*, which generalize ordinary graphs, have emerged as a viable alternative to model these networks (Klamt *et al.*, 2009). Hypergraphs correctly model a given reaction, which often has multiple input reactants and multiple output products, by a single *hyperedge* (that generalizes an ordinary graph edge), now directed from the set of reactants (its *tail*) to the set of products (its *head*). Positive regulation has been incorporated into hypergraph models by including enzymes in the tail of the hyperedge corresponding to the reaction they regulate; however, no hypergraph models in the literature address negative regulation.

A fundamental task in metabolic and cell-signaling networks is to find the most efficient series of reactions to synthesize a set of target molecules from the set of source compounds available to the organism or cell, while not exhausting any intermediate metabolites. This task maps to the *minimum-hyperedge factory* problem we consider here: Given a metabolic network represented by a directed hypergraph, together with the set of available source compounds, a set of target molecules, and the reaction stoichiometries, find a factory (a specialized pathway that takes into account stoichiometry of intermediate metabolites) that produces the targets from the sources using the fewest hyperedges, without interference from negative regulation. We give a brief overview of related work next.

### Related work

Previous methods for pathway inference in metabolic and cell-signaling networks have largely focused on three models: factories, elementary flux modes, and hyperpaths, which we summarize in turn.

Factories have been studied mainly in the context of the *minimum precursor* problem: Given a metabolic network represented by hypergraph *G*, a set of sources *S*, and a set of targets *T*, find a subset P⊆S, called the precursors, of minimum cardinality |P| that can produce the targets *T* via reactions in *G* without exhausting intermediate metabolites. Cottret et al. (2008) show the minimum precursor problem is NP-complete, while Zarecki *et al.* (2014) consider precursors of minimum molecular weight. Methods have been developed to enumerate *all* minimal precursor sets, either ignoring stoichiometry (Acuña *et al.*, 2012) or including it (Andrade *et al.*, 2016). None of these methods minimize the number of reactions used to produce the targets *T* from the precursors *P*.

*Elementary flux modes* (EFMs) in metabolic networks are minimal sets of reactions that conserve intermediate metabolites (which have no proper subset of reactions that also conserves them), without specified sources or targets (Zanghellini *et al.*, 2013). Most methods enumerate *all* elementary flux modes, which quickly becomes infeasible for genome-scale networks, whose number of EFMs is vast. de Figueiredo *et al.* (2009) avoid enumerating all EFMs by computing just the *k*-smallest EFMs via mixed-integer linear programming (MILP), but due to the minimality of EFMs can only allow a single target. Two discrete models of transcriptional regulation have also been considered: Jungreuthmayer and Zanghellini (2012) take all possible EFMs as input, and find the smallest number of gene knockouts that preserve desirable EFMs and exclude undesirable EFMs, where regulation is accommodated through constraints in an MILP that exclude biologically-infeasible EFMs; while Jungreuthmayer *et al.* (2015) add discrete regulatory rules to a generative approach for EFM enumeration.

*Hyperpaths* in directed hypergraphs are a generalization of source-sink paths in ordinary graphs that, unlike factories, require a strict ordering of their hyperedges. Italiano and Nanni (1989) show that finding a shortest hyperpath is NP-complete, even for acyclic hypergraphs. Gallo *et al.* (1993) explore multiple forms of connectivity in directed hypergraphs, and define length measures called additive cost functions for which shortest hyperpaths can be computed efficiently. In metabolic networks, Carbonell *et al.* (2012) give an algorithm that finds a source-sink hyperpath whenever one exists, irrespective of its length. In cell-signaling networks, Ritz and Murali (2014) and Ritz *et al.* (2017) were the first to solve *acyclic* shortest hyperpaths through a practical MILP, which Schwob et al. (2021) extend with reaction time-dependencies. To handle cycles, Krieger and Kececioglu (2021, 2022a,b) present the first efficient heuristic for *general* shortest hyperpaths, prove it finds optimal hyperpaths in singleton-tail hypergraphs, develop an algorithm for tractably generating all source-sink hyperpaths, and demonstrate through comprehensive experiments that the heuristic almost always finds optimal hyperpaths in real cell-signaling networks. These approaches address *positive regulation* by adding positive regulators to the tails of hyperedges for the reactions they regulate.

In contrast to prior work, we consider the new problem of finding a factory with the *fewest hyperedges* that produces *multiple targets* from the given sources. We also incorporate for the first time two discrete models of higher-order *negative regulation*: the first-order model captures direct interference among reactions within the factory, while the second-order model captures indirect interference from reactions outside the factory.

### Our contributions

We compute optimal factories that produce multiple targets from multiple sources using the minimum number of reactions, while for the first time incorporating negative regulation. More specifically, this work makes the following contributions.

- We introduce the new problem of finding a *minimum-hyperedge factory* that uses the fewest reactions to produce targets from sources.
- For the first time, we incorporate *negative regulation* into optimal factories, considering both its first- and second-order effects.
- We perform the first *comparative study* of pathway models: minimum-precursor factories versus shortest hyperpaths versus minimum-hyperedge factories with first- and second-order negative regulation.
- We prove that finding minimum-hyperedge factories is *NP-complete*, hence there is likely no method that finds optimal factories and is efficient in the worst-case.
- Even so, in practice our approach to optimal factories via mixed-integer linear programming is almost always remarkably *fast*, with a median run time of only a few seconds while including first- and second-order negative regulation, as demonstrated through *comprehensive experiments* across all instances from standard reaction databases.

A preliminary implementation of our method for optimal factories in a new tool we call Odinn (short for “optimal minimum-hyperedge factories in metabolic networks with negative regulation”), together with all datasets, is available free for non-commercial use at http://odinn.cs.arizona.edu.

### Plan of the paper

In the next section, we define optimal factories in metabolic networks, incorporate negative regulation, tackle the problem via mixed-integer linear programming, and show that finding minimum-hyperedge factories is NP-complete. Section 3 then presents experimental results, over all instances from the two standard reaction databases, comparing minimum-hyperedge factories to minimum-precursor factories and shortest hyperpaths, analyzes the effects of negative regulation, and demonstrates the practicality of finding optimal factories by our approach. Section 4 highlights the differences between solutions to these pathway models on concrete biological examples, and discusses the sensitivity of our model’s behavior to a critical input parameter. Finally Section 5 concludes, and offers directions for further research.

## 2 Methods

To present our methods for finding optimal metabolic factories, we first define versions of the minimum factory problem with or without negative regulation, and then explain how we reduce these formulations to solving an optimization problem known as a mixed-integer linear program.

### 2.1 Defining minimum factories in metabolic networks

Informally, a *factory* in a metabolic network is a collection of reactions that produce a set of target substances starting from a set of source substances, properly taking into account the stoichiometries of intermediate metabolites in reactions. The reactions in the factory may form cycles, and effectively can proceed simultaneously. (This is in contrast to the notion of a *hyperpath*, which is also a collection of reactions that produce the targets from the sources, but without taking into account stoichiometry, and where the reactions in the hyperpath must be ordered so that in each successive reaction all its input reactants must have been formed as output products of prior reactions in the ordering.) For factories, two relevant optimization criteria are: minimizing how many *sources* it uses to produce the targets, which in synthetic biology reduces the number of source substances that must be synthesized to produce the targets; or minimizing how many *reactions* it involves, which in pathway inference may yield a more likely pathway by which the cell produces the targets.

For the intermediate metabolites involved in a factory (the substances other than the sources and the targets), the stoichiometry ratios for the input reactants and output products of the factory’s reactions must be such that one of two conditions are met: either intermediate metabolites neither build up nor get depleted as the factory continues to produce the targets, known as *conservation*; or intermediate metabolites are allowed to build up, but not be depleted, known as *accumulation*. Under conservation or accumulation, by continuously supplying just the source substances to the factory, the targets can be produced indefinitely.

A key aspect to consider in a factory is *negative regulation*, where a substance participating in the factory may interfere by down-regulating a reaction in the factory, disrupting production of the targets. While negative regulation has not been incorporated into prior optimization formulations of factories, we categorize three levels of modeling its higher-order effects: (i) *zeroth-order*, which neglects it; (ii) *first-order*, which models direct negative regulation between the reactions in a factory; and (iii) *second-order*, which models indirect negative regulation, where a substance not participating directly in the reactions of the factory can still be produced indirectly from the factory’s sources via other reactions outside the factory, and end up negatively regulating reactions within the factory.

We next formally define the computational problem of finding optimal factories, under conservation or accumulation, taking into account zeroth-, first- and second-order negative regulation.

#### Formulating minimum factories without negative regulation

To properly represent the reactions in a metabolic network, where a given reaction can have multiple input reactants and multiple output products, requires a generalization of ordinary directed graphs known as a directed *hypergraph* G=(V,E), consisting of a set of directed *hyperedges E* corresponding to the reactions of the network, and a set of vertices *V* corresponding to the substances participating in the reactions. Each hyperedge e∈E is an ordered pair (*X*, *Y*) where both X,Y⊆V are nonempty sets of vertices, and *e* is directed from set *X* to set *Y*. Here, *X* is called the *tail* of *e*, and *Y* is called its *head*, given by functions tail(e)=X and head(e)=Y. We refer to the in-edges of a vertex v∈V by in(v)={e∈E:v∈head(e)}, and its out-edges by out(v)={e∈E:v∈tail(e)}. For a metabolic reaction represented in hypergraph *G* by hyperedge *e*, tail(e) is all the input reactants for the reaction, and head(e) is all its output products. For a reversible reaction in a metabolic network, we represent it in *G* by a pair of hyperedges e=(X,Y) and its reverse hyperedge rev(e)=(Y,X). Typically for a metabolic network represented by hypergraph *G*, the *sources* S⊆V of the network are the vertices with no in-edges, while *targets* T⊆V are often (but not always) vertices with no out-edges.

A key concept for metabolic factories is the notion of *flux*, which is the relative rate at which each reaction is used in its forward direction by the factory. In a hypergraph, we represent the flux for a factory by a nonnegative real-valued vector $f={\left(f_{e}\right)}_{e\in E}$, with all $f_{e}\;\geq \;0$. For a metabolic network represented by a hypergraph with n=|V| vertices and m=|E| hyperedges, the stoichiometry ratios of the substances in the reactions of the network can be summarized by an *n *×* m stoichiometry matrix* $M=(r_{ij})$, where *r_ij_* is the stoichiometry ratio for substance *i* in reaction *j*, or equivalently $r_{v,e}$ gives this ratio for vertex *v* in hyperedge *e*. For v∈tail(e), which correspond to an input reactant for the reaction, ratio $r_{v,e}$ is negative, representing that substance *v* is consumed in reaction *e*. For v∈head(e), corresponding to an output product of the reaction, ratio $r_{v,e}$ is positive, as *v* is produced by *e*. (If *v* is both in tail(e) and head(e), quantity $r_{v,e}$ is the difference between the reaction’s stoichiometry ratios for *v* as an output product and as an input reactant.) When v∈tail(e) and v∈head(e), in stoichiometry matrix $M=(r_{v,e})$ we assign $r_{v,e}=0$.

The utility of stoichiometry matrix *M* with respect to flux *f* for a factory is in capturing conservation or accumulation of intermediate metabolites. For a set I⊆V of intermediate metabolites, we denote by $M{|}_{I}$ matrix *M* restricted to its rows corresponding to *I*. Then the matrix–vector product $M{|}_{I}\cdot f$ is a vector giving for each intermediate metabolite v∈I the relative excess of *v* produced by the reactions in the factory under flux *f*. The condition $M{|}_{I}\cdot f=0$ corresponds to conservation, while $M{|}_{I}\cdot f\geq 0$ corresponds to accumulation.

Given flux *f* for a factory, we say a hyperedge *e* with $f_{e}>0$ is an *active edge*, meaning its corresponding reaction is used by the factory. Similarly, we say a source s∈S is an *active source*, meaning this source is used by the factory to produce the targets, if $\sum_{e\in \text{out}(s)}\;f_{e}>0$.

We now formally define the basic problem of finding a factory in a metabolic network that uses the fewest reactions, where we do not yet consider the effects of negative regulation. This basic problem has two versions below, according to whether we require conservation or accumulation of intermediate metabolites.

**Definition 1 (Minimum-Hyperedge Factory)** The *Minimum-Hyperedge Factory* problem without negative regulation is the following.

 The input is a metabolic network represented by hypergraph G=(V,E) with stoichiometry matrix *M*, candidate sources S⊆V, target molecules T⊆V−S, and minimum-flux constant ϵ>0.

The output is nonnegative flux *f* such that, for all intermediate metabolites I=V−(S∪T) either

- (conservation) $M{|}_{I}\cdot f=0$ or
- (accumulation) $M{|}_{I}\cdot f\geq 0$ holds,

the following production condition holds for each target t∈T,

$$\sum_{e\;\in \;\text{in}(t)}f_{e}\geq \epsilon ,$$

and the number of active edges $\left|\{e\in E:f_{e}>0\}\right|$ is minimum. ◻

This finds a metabolic factory, given by flux *f*, that produces all targets *T* from the sources *S* using the fewest reactions. We can also have edge weights, and minimize the total weight of the active edges.

The *Minimum-Source Factory* problem is the same as the above, except the objective is to instead minimize the number of active sources, $\left|\left\{s\in S:\sum_{e\in \text{out}(s)}\;f_{e}>0\right\}\right|$.

We use the shorter terms *min-edge factory* and *min-source factory* to refer to optimal solutions to these two problems. One advantage of Minimum-Hyperedge Factory over Minimum-Source Factory in practice is that a min-source factory can contain useless cycles that are disconnected from the sources and targets but circulate nonzero flux (as later illustrated in Figure 3), while this can never occur in a min-edge factory.

#### Including first-order negative regulation

We extend Minimum-Hyperedge Factory to include first-order negative regulation as follows. The input G,M,S,T,ϵ, output *f*, the conditions on *f*, and the minimization criteria are all the same, except now we also require that for flux *f* none of its active edges *e* are negatively regulated by any inhibitor *v* that is produced by another active edge *d* in the factory with v∈head(d). This captures the absence of direct negative regulation between reactions used by the optimal factory.

#### Including second-order negative regulation

To further extend Minimum-Hyperedge Factory to include second-order negative regulation, in addition to the first-order requirements given above, we also require that none of its active edges *e* are negatively regulated by any inhibitor *v* that can be produced from the active sources A⊆S of the factory. This captures the absence of indirect negative regulation of reactions used by the factory via inhibitors produced from the active sources by reactions outside the factory.

#### Complexity of computing optimal factories

We now show Minimum-Hyperedge Factory is NP-complete, so there is likely no algorithm that finds min-edge factories that is efficient in the worst-case. In the following, the *decision version* of the problem has an additional input parameter ℓ, and asks whether a given instance of the problem has a factory with at most ℓ active edges.

**Theorem 1 (NP-completeness)** *The decision version of Minimum-Hyperedge Factory under conservation is NP-complete.*

**Proof** We use a reduction from Exact Cover by 3-sets, which is NP-complete (Garey and Johnson, 1979, p. 221). Recall an instance of Exact Cover is a ground set *X* where |X|=n=3k, together with a family Y of subsets of *X*, where all subsets Y ∈ Y in the family have exactly |Y| = 3 elements. The problem is to determine whether there is a subfamily $\tilde{Y}\subseteq Y$ whose subsets $Y\in \tilde{Y}$ cover every element of *X* exactly once.

Given an instance X,Y of Exact Cover, we construct an instance G,M,S,T,ϵ,ℓ of Minimum-Hyperedge Factory under conservation as follows. Hypergraph *G* has a single source {s}=S, a single target {t}=T, vertices *v_Y_* for each family subset Y∈Y, and vertices *w_x_* for each ground-set element x∈X. The hyperedges of *G* are in three levels. The *top level* connects source *s* to the subset vertices *v_Y_* by ordinary edges $e=(s,v_{Y})$ with stoichiometry ratios $r_{s,e}=-1$ and $r_{v_{Y},e}=+3$. The *intermediate level* connects each of these subset vertices *v_Y_* to the corresponding ground-set vertices *w_x_* for each of its three elements x ∈ Y again by ordinary edges $e=(v_{Y},w_{x})$ with stoichiometry ratios $r_{v_{Y},e}=-1$ and $r_{w_{x},e}=+1$. Lastly, the *bottom level* connects all of these ground-set vertices *w_x_* to the target *t* conceptually by a large hyperedge whose head set consists of all *w_x_* and whose tail set contains only *t*. Our construction instead implements this large conceptual hyperedge for ground set $X=\{x_{1},\ldots ,x_{n}\}$ by a *daisy chain* of smaller hyperedges $e_{1},\ldots ,e_{n-1}$ involving additional vertices $u_{1},\ldots ,u_{n-2}$, where the first hyperedge $e_{1}=(\{w_{x_{1}},w_{x_{2}}\},\{u_{1}\})$, in general $e_{i}=(\{u_{i-1},w_{x_{i+1}}\},\{u_{i}\})$ for 1<i<n−1, and the last hyperedge $e_{n-1}=(\{u_{n-2},w_{x_{n}}\},\{t\})$, with stoichiometry ratios for all daisy-chain hyperedges *e_i_* of –1 for both tail vertices of *e_i_*, and ratio +1 for the single head vertex of *e_i_*. Finally, we use minimum-flux constant ϵ = 1, and active-edge upper-bound ℓ=7k−1.

We now claim X,Y is a yes-instance of Exact Cover iff G,M,S,T,ϵ,ℓ is a yes-instance of Minimum-Hyperedge Factory. For the forward implication, suppose $\tilde{Y}\subseteq Y$ is an exact cover for *X*. Consider a flux *f* that has unit flux on each edge in the top and intermediate levels of *G* that touches a vertex *v_Y_* for $Y\in \tilde{Y}$, unit flux on each hyperedge in the bottom daisy-chain level, and zero flux on all other edges. It is straightforward to verify flux *f* corresponds to a factory yes-instance.

For the reverse implication, suppose the constructed instance of Minimum-Hyperedge Factory is a yes-instance with corresponding flux *f*, and rescale *f* so it has exactly unit flux entering target *t*. Conservation at all vertices *u_i_* implies every edge in the daisy chain also has unit flux. Thus for all 3*k* elements x∈X, vertex *w_x_* has outgoing unit flux, so conservation at all *w_x_* implies at least 3*k* edges at the intermediate level must be active. Since each vertex *v_Y_* has only three out-edges at the intermediate level, conservation at all *v_Y_* implies a lower bound of at least *k* active edges at the top level. On the other hand, we have an upper bound of at most *k* active edges at the top level, since in total *f* has at most ℓ=7k−1 active edges, while there are 3k−1 active edges in the daisy chain, and at least 3*k* active edges at the intermediate level. Combining both bounds, the top level has exactly *k* active edges. These *k* active top-level edges touch subsets *Y* that form an exact cover of *X*, demonstrating this is also a yes-instance.

Finally, the instance of Minimum-Hyperedge Factory in the reduction can be constructed from X,Y in polynomial time, so the problem is NP-hard. Moreover it is in NP, as after non-deterministically guessing at most ℓ active edges, we can find a flux *f* that satisfies conservation and produces target *t* in polynomial time by solving a linear programming problem that enforces zero flux on all non-active edges. Thus it is NP-complete. ◻

We note this proof shows Minimum-Hyperedge Factory is NP-complete as well when including first- and second-order *negative regulation* (since the instance constructed in the reduction has no negative regulators). Furthermore, the proof also shows the problem is NP-complete when the hypergraph is *acyclic*, has a *single source* and a *single target*, and when every hyperedge has only *one head-vertex* and at most *two tail-vertices*. A minor modification to the proof shows the problem remains NP-complete under *accumulation* as well.

### 2.2 Finding factories via integer linear programming

We now show that all versions of Minimum-Hyperedge Factory (with conservation or accumulation, and none, first- or second-order negative regulation) can be reduced to solving either a single instance, or a series of instances, of a constrained linear optimization problem known as a *mixed-integer linear program* (MILP). An MILP optimizes a linear function of a collection of variables, some of which are real-valued and others integer-valued, subject to constraints that are linear inequalities in the variables.

#### Modeling minimum factories without negative regulation

We model Minimum-Hyperedge Factory without negative regulation as an MILP as follows. An instance of Min-Edge Factory consists of hypergraph G=(V,E), candidate sources S⊆V, targets T⊆V−S, stoichiometry matrix *M*, and constant ϵ>0. We next describe the variables, constraints, and objective function of the corresponding MILP for this instance.

The *variables* are in two groups. Flux vector $f={\left(f_{e}\right)}_{e\in E}$ consists of real-valued variables *f_e_*, and active-edge vector $x={\left(x_{e}\right)}_{e\in E}$ consists of integer-valued variables *x_e_*.

The *constraints* are in three classes. The domain constraints are $0\leq f_{e}\leq 1$ and $0\leq x_{e}\leq 1$ for all hyperedges e ∈ E (which ensures $x_{e}\in \{0,1\}$). For the intermediate metabolites I=V−(S∪T), we have either the conservation constraints $M{|}_{I}\;\cdot \;f=0$, or the accumulation constraints $M{|}_{I}\;\cdot \;f\geq 0$. For each target molecule t∈T, the production constraint $\sum_{e\in \text{in}(t)}\;f_{e}\geq \epsilon$ ensures target *t* is produced. For hyperedges e∈E, the active edge constraints $x_{e}\geq f_{e}$ ensure *x_e_* = 1 for an active edge *e* with $f_{e}>0$. Lastly for pairs of reverse hyperedges *e* and rev(e) that model a single reversible reaction, the reversible-reaction constraints $x_{e}+x_{\text{rev}(e)}\leq 1$ prevent trivial cycles that send flux through both *e* and its reverse.

The *objective function* is to minimize $\sum_{e\in E}\;x_{e}$. Notice that for this objective, an optimal solution $f^{*},x^{*}$ has $x_{e}^{*}=1$ iff $f_{e}^{*}>0$, so this MILP captures the problem of finding a factory that produces the targets *T* with the minimum number of active edges.

We can model Minimum-Source Factory without negative regulation by a similar MILP. Briefly, we now have an active-source vector $y={\left(y_{s}\right)}_{s\in S}$ with integer variables $y_{s}\;\in \;\{0,1\}$, where for every candidate source s∈S and every hyperedge e∈out(s), we have the active-source constraints $y_{s}\geq f_{e}$. The objective function is then to minimize $\sum_{s\in S}\;y_{s}$, the number of active sources.

We can also combine these two MILPs for Min-Edge Factory and Min-Source Factory into a single MILP whose variables include both active-edge vector *x* and active-source vector *y*, that contains the union of their constraints, and minimizes a linear combination of their objective functions. This has the advantage of solving one optimization problem that, by adjusting the weighting constant in the objective on the contribution of active edges versus active sources, yields a solution to the bicriteria problem of finding a min-edge factory that uses the fewest sources, or a min-source factory that has the fewest edges, as discussed in Section 3.2.

Finally, we mention the value of minimum-flux constant ϵ > 0, that ensures targets *T* are produced, is critical. Since for any flux vector *f* that satisfies conservation or accumulation the scaled flux vector cf for positive constant *c* also satisfies these conditions, we can always rescale solutions so that each $f_{e}\in [0,1]$. Nevertheless, determining *a priori* a good lower bound on how small the non-zero flux on active edges can get in an optimal solution after such rescaling is challenging, as discussed in Section 4.2.

#### Modeling first-order negative regulation

We can easily accommodate first-order negative regulation within the above MILP. For every hyperedge e ∈ E that is negatively regulated by substance v ∈ V, and for all hyperedges d ∈ in(v) that produce *v*, we add the negative-regulation constraints $x_{e}+x_{d}\leq 1$. Since hyperedges *e* and *d* now cannot both be active, this prevents as a solution any factory that both uses a reaction *e* and directly negatively inhibits *e*.

#### Handling second-order negative regulation

For Minimum-Hyperedge Factory under second-order negative regulation, we want a min-edge factory whose active edges are not negatively regulated by any inhibitor that can be produced from its active sources. This complex constraint cannot be readily incorporated into the original MILP directly. Instead, we take an iterative approach that essentially generates next-best factories until finding one that satisfies second-order negative regulation.

Our algorithm first solves problem $P_{1}$, the original MILP above for Minimum-Hyperedge Factory under first-order negative regulation, to obtain initial solution $f^{\left(1\right)}$. In general at iteration *i*, it checks whether the current solution $f^{\left(i\right)}$ satisfies second-order negative regulation, as follows. Determine the active sources A⊆S of $f^{\left(i\right)}$, and for each active edge *e* of $f^{\left(i\right)}$ that has a negative regulator *v*, check whether *v* can be produced from *A* by setting up a linear program (LP) with only flux variables whose constraints include conservation or accumulation, that sets all fluxes to zero on out-edges of non-active sources, and instead of the target constraint has the production constraint $\sum_{e\in \text{in}(v)}\;f_{e}\geq \epsilon$. If this LP is feasible, inhibitor *v* can be produced from the active sources *A*, so $f^{\left(i\right)}$ is not second-order valid, and the process continues to the next iteration i+1 below. Otherwise, if no inhibitor of an active edge can be produced from the active sources, the algorithm halts, and outputs optimal factory $f^{\left(i\right)}$.

At iteration i+1, the algorithm adds two new constraints to the current MILP, which rule out the prior solution $f^{\left(i\right)}$, to obtain the next problem $P_{i+1}$. The first constraint requires, for the active edges F⊆E of $f^{\left(i\right)}$, that $\sum_{e\in F}\;x_{e}\leq |F|-1$. This constraint ensures that solving $P_{i+1}$ yields an optimal factory distinct from $f^{\left(i\right)}$, and by extension all prior factories. The second constraint requires, for hyperedge *e* that was negatively regulated by inhibitor *v* produced from the active sources A⊆S of $f^{\left(i\right)}$, that $x_{e}+\sum_{s\in A}y_{s}\leq |A|$. This constraint ensures that solving $P_{i+1}$ yields an optimal factory that does not both contain active edge *e* and use the same active sources *A* (since if it does, hyperedge *e* will again be negatively regulated by *v*). The algorithm then solves problem $P_{i+1}$ and repeats.

This solves a series of MILPs $P_{1},P_{2},\ldots$ until finding the optimal factory satisfying second-order negative regulation. In practice, this algorithm is remarkably fast for the vast majority of instances from standard reaction databases, as discussed in Section 3.4.

## 3 Results

We now present results from computational experiments on real biological datasets comparing the structure of min-edge factories, min-source factories, and shortest hyperpaths. We also study the effects of negative regulation on optimal factories, and evaluate the speed of our optimization-based approach.

### 3.1 Experimental setup

We first detail the datasets used in our experiments, and then describe the implementation of our methods.

#### Datasets

We evaluate our approach to min-edge factories on eight standard datasets. Seven of these datasets are metabolic networks for different organisms taken from MetExplore (Cottret *et al.*, 2018): namely, *Buchnera aphidicola*, *Baumannia cicadellinicola*, *Carsonella ruddii*, *Escherichia coli*, *Homo sapiens*, *Saccharomyces cerevisiae*, and *Sphenomorphus muelleri*. We identify these datasets by an abbreviation of the organism name (first letter of genus, underscore, first three letters of species). These metabolic datasets were downloaded as SBML files, and parsed into hypergraphs. We note that these seven datasets do not contain any regulation information.

The eighth and largest dataset comes from Reactome (Joshi-Tope *et al.*, 2005), which contains curated human signaling pathways. To build the Reactome dataset, we downloaded all Reactome pathways in BioPAX format (Demir *et al.*, 2010) from Pathway Commons, concatenated them together, and formed the hypergraph using a modified version of a parser from Franzese *et al.* (2019). Reactome includes 6,051 reactions and 483 reactions annotated with positive and negative regulators, respectively.

For each dataset, we constructed a hypergraph by mapping each entity (protein, protein complex, and so on) to a vertex in the hypergraph, accounting for different compartmentalization and post-translational modification. (So the same protein in the nucleus and cytoplasm, for example, is represented by two different vertices.) This is because many of the pathways specifically describe protein transport between cellular compartments, or post-translational modifications. We mapped each reaction to a unit-weight hyperedge, with reactants in the tail of the hyperedge, and products in the head. Following the precedent from Ritz *et al.* (2017), positive regulators were added to the tails of hyperedges they regulate. Negative regulators and stoichiometry ratios for each hyperedge were stored separately, where unit stoichiometry ratios were used when they were missing. Reversible reactions were modeled as two separate hyperedges with their heads and tails reversed.

We consider any vertex with no in-edges a *source*, and any vertex with no out-edges a *target*. A problem *instance* then involves finding a factory (or hyperpath) from all of the sources to a given target. (When computing hyperpaths, we created a supersource, and a zero-weight hyperedge with the supersource as its tail, and all source vertices in its head; we also added to its head any vertices whose only in-edge is a self-loop, as otherwise these self-loops are unreachable.)

Table 1 gives statistics on the hypergraphs constructed for each dataset, listed in order of increasing size. Overall, the hypergraphs are very sparse, having fewer hyperedges than vertices in half the datasets. While the hypergraphs do contain a few highly-connected vertices representing ubiquitous molecules, most vertices have very low connectivity, in terms of the small median in- and out-degrees (as well as the small median hyperedge tail and head sizes).

**Table 1.** Dataset summaries

	C_Rud		S_Mue		B_Aph		B_Cic		S_Cer		H_Sap		E_Col		Reactome	
Vertices	263		314		460		700		936		1618		1877		20 458	
Hyperedges	229		273		447		755		1250		2132		2999		11 802	
Sources	40		45		45		58		128		171		65		8296	
Targets	44			48		51		67		227		344		73	5066	
	median	max	median	max	median	max	median	max	median	max	median	max	median	max	median	max
Tail size	2	4	2	4	2	6	2	6	1	2	1	2	2	7	2	26
Head size	2	5	2	5	2	6	2	6	1	3	1	3	2	95	1	28
In-degree	1	41	1	49	1	67	1	156	1	15	1	13	1	806	1	1056
Out-degree	1	64	1	72	1	104	1	142	1	8	1	18	1	511	1	1167

#### Implementation

Our new tool Odinn (Krieger and Kececioglu, 2022c) implements both min-edge and min-source factories, comprising around 300 lines of Python code. The parser to convert the BioPAX format into hypergraphs is from Franzese *et al.* (2019) and was modified to include stoichiometry and negative regulators. The shortest acyclic hyperpath MILP is from Ritz *et al.* (2017). Halp (https://github.com/Murali-group/halp) was used for directed hypergraph representations. MILPs were solved using CPLEX 12.6, run on an M1 processor with 8 Gb of memory.

### 3.2 Comparing min-edge factories to current models

We highlight the advantages of min-edge factories by comparing them to the current alternate models of min-source factories (Andrade *et al.*, 2016) and shortest acyclic hyperpaths (Ritz *et al.*, 2014).

*Shortest acyclic hyperpaths* Hyperpaths have strict order requirements on their hyperedges that factories do not, so instances with a factory may not have a hyperpath. As Table 2 shows, there are generally more instances with factories than hyperpaths. In the seven metabolic datasets, when hyperpaths exist they tend to be very short, sometimes with only one hyperedge. Table 3 shows that in Reactome, the length of the shortest acyclic hyperpath and min-edge factory differ on just 2% of the instances under accumulation, and only 6% under conservation. The vast majority of Reactome instances likely contain a shortest hyperpath that is also a min-edge factory under accumulation due to many reactions having unit stoichiometry. An instance where the shortest acyclic hyperpath is longer than the min-edge factory is discussed later in Section 4.1.

**Table 2.** Target instance feasibility

	C_Rud	S_Mue	B_Aph	B_Cic	S_Cer	H_Sap	E_Col	Reactome
Target instances	44	48	51	67	227	344	73	5066
Instances with factory under accumulation	12	23	34	39	169	320	58	3955
Instances with factory under conservation	2	1	3	13	160	265	1	1649
Instances with acyclic hyperpath	1	2	2	1	165	312	1	2432

*Min-source factories* Min-edge factories tend to also minimize their number of sources. Table 3 shows the fraction of instances where the min-edge and min-source factory have the same number of sources or hyperedges. The number of sources agrees for both factories under conservation on most instances, possibly due to there being few feasible factories per instance. Even under accumulation, their median number of sources is close. 

**Table 3.** Solution structure

	C_Rud		S_Mue		B_Aph		B_Cic		S_Cer		H_Sap		E_Col		Reactome	
	acc.	cons.	acc.	cons.	acc.	cons.	acc.	cons.	acc.	cons.	acc.	cons.	acc.	cons.	acc.	cons.
Sources same number (min-edge, min-source)	41%	100%	35%	100%	50%	100%	10%	100%	51%	51%	65%	64%	14%	100%	23%	99%
Hyperedges same number (min-edge, min-source)	33%	100%	0%	0%	0%	33%	3%	0%	0%	50%	0%	59%	2%	0%	0%	96%
Hyperedges same number (min-edge, hyperpath)	100%	0%	100%	100%	100%	100%	100%	100%	93%	66%	92%	88%	100%	100%	98%	94%
	med.	max	med.	max	med.	max	med.	max	med.	max	med.	max	med.	max	med.	max
Sources (min-edge, accumulation)	2	6	3	7	3	6	3	7	1	7	1	4	4	7	2	43
Sources (min-source, accumulation)	2	5	1	4	2	4	2	4	1	4	1	3	1	2	2	40
Sources (min-edge, conservation)	1	1	1	1	1	1	2	2	1	4	1	3	1	1	2	20
Sources (min-source, conservation)	1	1	1	1	1	1	2	2	1	4	1	2	1	1	2	20
Hyperedges (min-edge, accumulation)	4	7	6	22	5	15	4	30	3	48	3	44	13	90	2	37
Hyperedges (min-source, accumulation)	5	10	27	40	10	27	10	62	12	61	9	108	163	205	3	39
Hyperedges (min-edge, conservation)	4	5	3	3	3	7	7	14	3	48	3	44	1	1	1	24
Hyperedges (min-source, conservation)	4	5	6	6	8	8	10	34	5	54	3	99	100	100	1	25
Hyperedges (hyperpath)	1	1	1	1	1	2	2	2	3	48	3	44	1	1	1	16

On the other hand, min-source factories tend to use much more than the minimum number of reactions. Table 3 shows the number of hyperedges in the two types of factories under accumulation differs significantly on nearly all instances. Min-source factories can contain useless cycles that do not contribute to target production, as later illustrated in Section 4.1.

The merits of min-edge factories are also shown when considering bicriteria optimization (as mentioned earlier in Section 2.2). We computed the min-edge factory with fewest sources, the min-source factory with fewest hyperedges, and compared them to standard min-edge and min-source factories. On over 95% of Reactome instances, the number of hyperedges differs between the standard min-source factory and its bicriteria version having fewest hyperedges (with a median difference of 1 hyperedge, and a maximum difference of 37). In contrast, on less than 1% of Reactome instances, the number of sources differs between the standard min-edge factory and its bicriteria version having fewest sources (with a median difference of 3 sources, and a maximum difference of 10). In brief, min-edge factories almost always minimize their number of sources as well—while the opposite is not true for min-source factories.

### 3.3 Effects of negative regulation on optimal factories

For a given instance, the hyperedges in the optimal min-edge factory without negative regulation (zeroth-order factories) often differ from the hyperedges in the optimal min-edge factory satisfying higher-order negative regulation (first- and second-order factories). In fact for many Reactome instances, all feasible factories violate higher-order negative regulation.

Table 4 gives the number of Reactome instances with a feasible factory for each order of negative regulation, along with statistics on instances with longer min-edge factories under negative regulation. (We note that Reactome is the only dataset with negative regulation information.) Under accumulation, 22 instances have no first-order factories, while for 83 instances all optimal zeroth-order factories violate first-order negative regulation. This clearly demonstrates the importance of directly considering negative regulation in pathway inference.

**Table 4.** Negative regulation feasibility and structure

	Reactome			
	accumulation		conservation	
0th-order factory instances (min-edge and min-source)	3955		1649	
1st-order factory instances (min-edge and min-source)	3933		1640	
2nd-order factory instances (min-edge)	3671		1614	
Instances where 1st- worse than 0th-order (min-edge)	83		6	
Instances where 1st- worse than 0th-order (min-source)	14		0	
Instances where 2nd- worse than 1st-order (min-edge)	11		4	
	median	max	median	max
0th- to 1st-order factory length change	1	4	2	3
0th- to 1st-order factory source change	1	4	0	0
1st- to 2nd-order factory length change	3	6	3	4
Number of 2nd-order iterations	1	554	1	285

Even more instances have zeroth-order factories that violate second-order negative regulation. Under accumulation, a further 262 instances have no second-order factories, while for 11 instances all optimal first-order factories violate second-order negative regulation. In addition, for these 11 instances, the optimal second-order factory is often much longer than the optimal first-order factory.

**Fig. 1.**
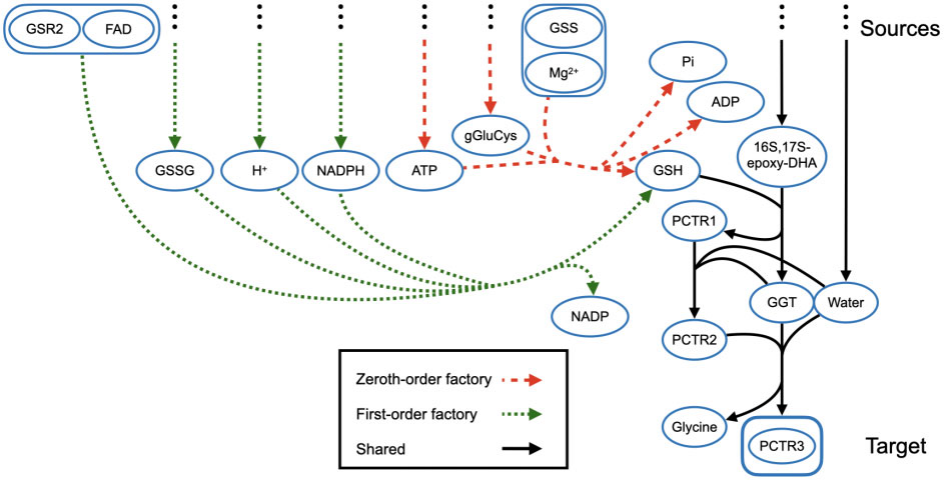
Min-edge factories under zeroth- and first-order negative regulation to the target “protectin conjugate in tissue regeneration 3” (PCTR3) from Reactome. The solid black hyperedges appear in both the optimal min-edge factory without negative regulation (zeroth-order) and the min-edge factory under first-order negative regulation. Hyperedges in red with a longer dash are only in the zeroth-order factory, while hyperedges in green with a shorter dash are only in the first-order factory. Some hyperedges from the sources have been replaced with ellipses to simplify the drawing. The zeroth-order and first-order factories have 19 and 21 hyperedges, respectively. The single hyperedge in longer red dash that is shown is internally negatively regulated within the zeroth-order factory, and its role is effectively replaced in the first-order factory by the single hyperedge in shorter green dash that is shown. The min-edge factory under second-order negative regulation again has 21 hyperedges, but also differs from the first-order factory by a single hyperedge, due to external negative regulation.

We now give two concrete biological examples that highlight the importance of discrete negative regulation in pathway inference. The first example is for the instance with the target “protectin conjugate in tissue regeneration 3” (PCTR3) from Reactome. Figure 1 illustrates the optimal zeroth- and first-order min-edge factories for this instance, where only a portion of these factories is explicitly drawn due to their complexity. The solid black hyperedges are common to both the zeroth- and first-order factories. The hyperedges in red with a longer dash are unique to the zeroth-order factory, while the hyperedges in green with a shorter dash are unique to the first-order factory. Several hyperedges have been replaced with ellipses to simplify the figure. Both factories contain hyperedges from the Reactome pathway “Biosynthesis of DHA-derived sulfido conjugates”, representing reactions that create PCTR3, a sulfido conjugate. Docosahexaenoic acid (DHA)—which appears in both factories—is an omega-3 fatty acid often found in fish. DHA is transformed through a cascade of reactions to a group of resolvins and protectins in response to inflammation (Molfino *et al.*, 2017). The zeroth-order factory has 19 hyperedges, and contains the hyperedge drawn with a red dash, which is a negative feedback loop where reduced glutathione (GSH) negatively regulates the hyperedge that produces it. The optimal first-order factory cannot use this hyperedge, and instead requires 21 hyperedges, which replace this hyperedge by a series of reactions that culminate in the green hyperedge shown. The optimal second-order factory also has 21 hyperedges, but now replaces an invalid upstream hyperedge whose negative regulator is reachable from the sources.

Our second example highlights how much the reactions utilized for a given instance can differ between the optimal zeroth- and first-order factories. For this example, we chose the instance from Reactome with Exoin E4 as a target, as it has the largest variation in number of hyperedges between the optimal zeroth- and first-order factories, with 21 and 25 hyperedges respectively. We first enumerated all optimal zeroth- and first-order factories. We then compared the hyperedges in each zeroth-order factory to each first-order factory, measuring their symmetric difference and percent overlap (namely the size of their intersection divided by their average length). The minimum symmetric difference was 10 (and must be at least 4 due to the length difference between factories), with a maximum of 26. The percent overlap between pairs ranged from 43% to 78% (so at most 78% of the hyperedges in every optimal zeroth-order factory appeared in any optimal first-order factory).

As this example demonstrates, optimal factories that ignore negative regulation can differ markedly from optimal factories that take into account even just first-order negative regulation.

### 3.4 Speed of computing optimal factories

Our MILP-based approach to *min-edge factories* with zeroth- and first-order negative regulation runs in a matter of seconds, with a median running time over all instances from all datasets of 3 seconds, and a maximum of 61 seconds. Finding *min-source factories* with zeroth- and first-order negative regulation is also fast, with a maximum running time of 10 seconds. Finding *shortest acyclic hyperpaths* is typically fast as well, but can get expensive: on the larger E_Col and Reactome datasets, its median running time is 10 seconds, while the maximum time is 5335 seconds, or nearly 15 hours.

Finding min-edge factories with *second-order negative regulation* is usually remarkably fast as well, with a median running time of 3.5 seconds. On instances, though, where no factory satisfies second-order negative regulation, it can end up generating all first-order factories that produce the target in order of increasing length, before finally failing. While the MILP is fast on the majority of these instances, there are twelve such instances that run longer than 6 hours, with a maximum running time of 70 hours.

## 4 Discussion

We next discuss two biological examples that compare minimum-hyperedge factories to minimum-source factories and shortest acyclic hyperpaths, and then examine the sensitivity of our model to the value of minimum-flux constant *ϵ*.

### 4.1 Comparing models on concrete examples

**Fig. 2.**
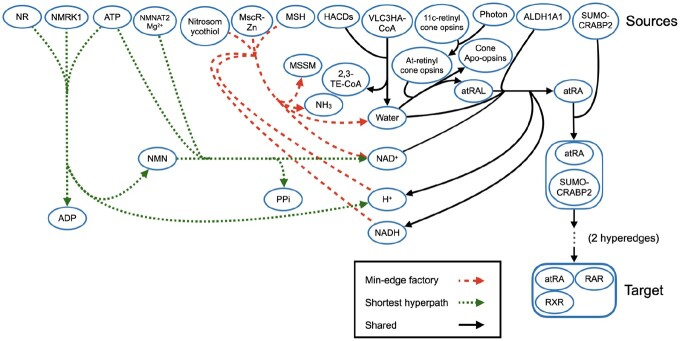
Minimum-hyperedge factory and shortest acyclic hyperpath to the atRA/RAR/RXR complex in Reactome, including positive but not negative regulation. Hyperedges in solid black appear in both the minimum-hyperedge factory and the shortest acyclic hyperpath. Hyperedges with a shorter green dash appear only in the shortest acyclic hyperpath, while hyperedges with a longer red dash only appear in the minimum-hyperedge factory. To simplify the drawing, two hyperedges creating the RAR/RXR/SUMO-CRABP2/atRA complex have been omitted.

**Fig. 3.**
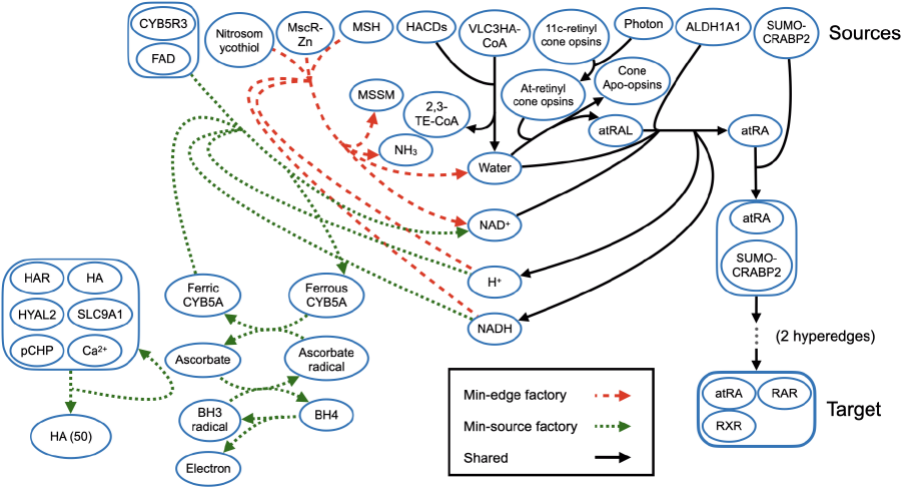
Minimum-hyperedge factory and minimum-source factory to the atRA/RAR/RXR complex in Reactome, including positive but not negative regulation. Hyperedges in solid black appear in both the minimum-hyperedge and minimum-source factory. Hyperedges with a shorter green dash appear only in the minimum-source factory, while hyperedges with a longer red dash only appear in the minimum-hyperedge factory. To simplify the drawing, two hyperedges creating the RAR/RXR/SUMO-CRABP2/atRA complex have been omitted.

Figures 2 and 3 contrast the optimal min-edge factory for a given instance with the shortest acyclic hyperpath (Fig. 2) and the optimal min-source factory (Fig. 3). Hyperedges common to all three pathway models are drawn in solid black, while hyperedges unique to the min-edge factory are drawn in a longer red dash, and hyperedges unique to the shortest hyperpath or min-source factory are drawn in a shorter green dash. (Some hyperedges common to both the factories and the hyperpath were removed to simplify the drawings.)

These figures show the metabolism of vitamin A (retinol) in Reactome pathway “Signaling by retinoic acid”. The pathway creates all-trans-retinal (atRAL) in the eye before being oxidized by aldehyde dehydrogenase 1A1 tetramer (ALDH1A1), forming all-trans-retinoic acid (atRA), which is free to bind with cellular retinoic acid-binding protein 2 (SUMO-CRABP2). It then moves to the nucleoplasm, where it binds with the retinoic acid receptor complex (RAR/RXR), and SUMO-CRABP2 disassociates, creating the target atRA/RAR/RXR complex.

Figure 2 reveals how hyperpaths can have more restrictive constraints than factories. Notice that in the factory, vertex H${}^{+}$ in the red-dashed hyperedge’s tail has not been reached by prior hyperedges. The shortest acyclic hyperpath cannot use this red hyperedge due to strict ordering requirements, and hence requires an additional hyperedge in its pathway.

Figure 3 illustrates how min-source factories can contain useless cycles that do not contribute to creating the target, such as the self-loop between the six-member complex and hyaluronic acid (HA). This extra cycle does not affect the active sources and so was included, as the min-source criterion does not attempt to minimize the number of hyperedges. The min-source factory also uses extra hyperedges (unrelated to the retinol pathway) to create NAD${}^{+}$, in order to minimize the number of active sources.

### 4.2 Sensitivity to the minimum-flux constant

The value of minimum-flux constant *ϵ* in our min-edge factory MILP is crucial. If *ϵ* is too large, factories that produce the target with non-zero flux smaller than *ϵ* are excluded as infeasible, even though they are valid solutions. If *ϵ* is too small, numerical errors can occur internally within the MILP solver, leading to outputs that are not actually valid factories.

We experimentally demonstrate the sensitivity to *ϵ* on a Reactome instance where we ran the min-edge MILP with three different values of *ϵ*: specifically $10^{-2},\;10^{-4}$, and $10^{-6}$. Even though this smallest value for *ϵ* is larger than the smallest real-value allowed by CPLEX (our MILP solver), the solution contained no active edges, and so was clearly not a valid factory. Factories for the two larger *ϵ*-values differed in their number of hyperedges, indicating the largest *ϵ*-value was too big and excluded valid factories. (This shows the necessity of validating factories output by the MILP.) In general, an *ϵ* of $10^{-4}$ did not return an invalid factory for any instance—though it is possible that a smaller *ϵ* could result in a valid factory with fewer hyperedges (that was excluded by this greater *ϵ*).

## 5 Conclusion

We have introduced the new problem of minimum-hyperedge factories, established its computational complexity, incorporated for the first time higher-order effects of negative regulation, computed optimal solutions via mixed-integer linear programming, and demonstrated the practicality of our approach through comprehensive experiments on real metabolic networks. Extensive comparisons with the current pathway models of minimum-source factories and shortest acyclic hyperpaths reveal structural differences between their solutions that highlight the value of computing min-edge factories with discrete negative regulation.

### Further research

The *minimum-flux constant ϵ* is crucial for our model, and an algorithmic approach for computing the largest valid *ϵ* for a given instance would be useful. Incorporating *third-order negative regulation* to model mutual interference between negative regulators that ultimately inhibit each other would better capture actual negative regulation in real metabolic networks.
